# Proteus Syndrome: Case Report and Updated Literature Review

**DOI:** 10.1055/a-2300-7002

**Published:** 2024-06-14

**Authors:** Maria K Klimeczek-Chrapusta, Marek Kachnic, Anna Chrapusta

**Affiliations:** 1 Malopolska Burn and Plastic Surgery Center, Ludwik Rydygier Memorial Hospital in Krakow, Cracow, Poland; 2 Student Scientific Group of Pediatric Surgery, Department of Pediatric Surgery, Jagiellonian University Medical College, Faculty of Medicine, Cracow, Poland

**Keywords:** case report, genetic disorders, Proteus syndrome, pediatric plastic surgery

## Abstract

Proteus syndrome (PS) is an exceptionally uncommon genetic disorder that has been documented in only approximately 250 cases in the literature spanning the past four decades. It is characterized by a disproportionate, asymmetric overgrowth of all types of tissues, provoked by a somatic activating mutation in serine/threonine protein kinase 1. We report a case of PS in a two-year-old female patient with the following clinical features: unilateral overgrowth of connective tissue in the right buttock and right foot, where multiple surgeries were performed to achieve a desirable aesthetic outcome and ensure psychological comfort of the young patient. The insights provided by this case underscore the pivotal role of obtaining pleasing aesthetic outcomes in the surgical management of untreatable genetic disorders, with the aim of nurturing psychological contentment in affected children.

## Introduction

Proteus syndrome (PS) is an extremely rare genetic hamartomatous disorder characterized by a disproportionate, asymmetric overgrowth with skeletal deformations, vascular malformations, and dysregulated adipose tissue. 1 It is provoked by a somatic activating mutation in serine/threonine protein kinase 1 (AKT1), causing mutation in the chimeric cells. 2 This activation limits apoptosis and promotes growth among other effects.

It was first described by Cohen and Hayden in 1979. 3 A few years later, in 1983, Hans-Rudolf Wiedemann, a German pediatrician named it after sea god Proteus, who could change his shape to evade capture. 4 5 The prevalence of this syndrome is believed to be less than 1:1,000,000. 6 Symptoms can manifest in various parts of the body and commonly commence during infancy. 7 They primarily revolve around skeletal overgrowth, yet this disorder exhibits significant pleiotropy, encompassing central nervous system overgrowth, neuronal migration abnormalities, vascular anomalies, overgrowth of various other organs and tissues, and the development of bullous or cystic lung diseases. 8 This syndrome itself is not inherited and does not pass to the offspring, however the life expectancy is short, due to many complications, most common being deep vein thrombosis, as a result of large venous capillary malformations. 5

With fewer than 250 cases documented in the literature, the rarity of its occurrence provides justification for this report. 9 This is the case report of a 22-month-old female patient who presented herself at the plastic surgery clinic with a rare case of PS, confirmed by genetic testing. The primary objective of our study is to present the results of the highly radical surgical resection of the overgrown tissue in this rare genetic disorder.

## Case

A 2-year-old female patient was presented at a plastic surgery clinic with the following clinical features: unilateral overgrowth of connective tissue in the right buttock and the right foot, where overgrowth of bone tissue was also found. When the child was 22 months old, the patient's parents complained of a systematic growth of the right buttock and difficulty in finding fitting shoes for the patient's right foot.

The parents of the patient affirmed that there was no family history of genetic disorders. The patient underwent genetic testing at the age of 20 months with a positive result for a somatic embryonal mutation of *AKT1* gene. Due to a high risk of deep vein thrombosis and embolism, additional genetic investigations were conducted ( Table 1 ). Upon detecting a heterozygous mutation in methylenetetrahydrofolate reductase enzyme gene ( *MTHFR* ), the patient was recommended for an annual screening of blood homocysteine levels. Furthermore, the parents were informed about their child's heightened need for vitamin B12 and folic acid.

**Table 1:** Assessed gene mutations and their clinical significance

Gene	Analyzed mutation	Clinical significance	Results
***Factor 2***	Prothrombin gene mutation 20210G > A	A mutation in this gene increases the risk for deep vein thrombosis, arterial thrombosis, ischemic stroke, and myocardial infraction, due to increased prothrombin production.	Negative
***Factor 5***	Arg534Gln (R506Q, factor V Leiden)	A mutation in this gene alters the composition of coagulation cascade factor V, rendering it resistant to the anticoagulant protein C. This mutation elevates the risk of deep vein thrombosis, arterial thrombosis, ischemic stroke, and myocardial infarction.	Negative
***MTHFR***	Ala222Val (C677T)	A mutation in this gene results in elevated levels of homocysteine in blood, potentially raising the susceptibility to cardiovascular diseases and hypercoagulability. In contrast to homozygous mutations, heterozygous mutations often present as asymptomatic.	Positive (heterozygous)

Abbreviation: MTHFR, methylenetetrahydrofolate reductase enzyme.

**Fig. 1:**
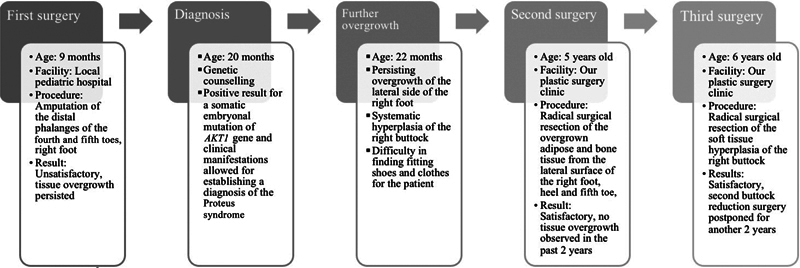
Chart presenting the surgical procedures that the patient underwent.

When the patient was 9 months old, they underwent a surgical procedure at another facility to amputate the distal phalanges of the fourth and fifth toes due to overgrowth. As time progressed, tissue overgrowth persisted, and the aesthetic results of the surgery remained unsatisfactory ( Fig. 1 ).

Physical examination and radiological screening prior to the correction surgery did not reveal any additional abnormalities. Patient's parents gave an informed consent for the surgery of their child.

**Fig. 2:**
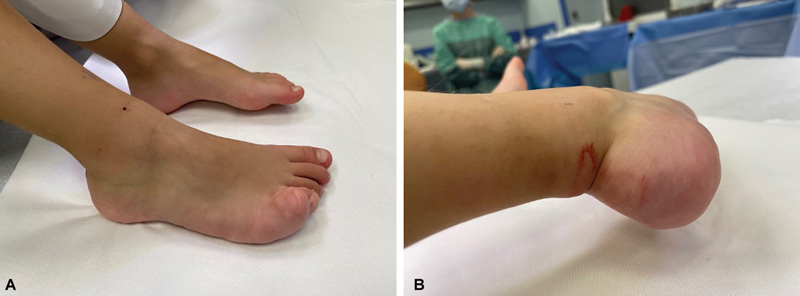
Photos of the patient's feet taken before the first tissue reduction surgery at our clinic, after the initial amputation surgery of distal phalange of fourth and fifth toes, performed at a different facility. ( **A** ) The picture of the overgrown tissues on the lateral side of the right foot, mostly prevalent in the fourth and fifth toes. ( **B** ) The picture of the overgrown tissues of the right heel.

**Fig. 3:**
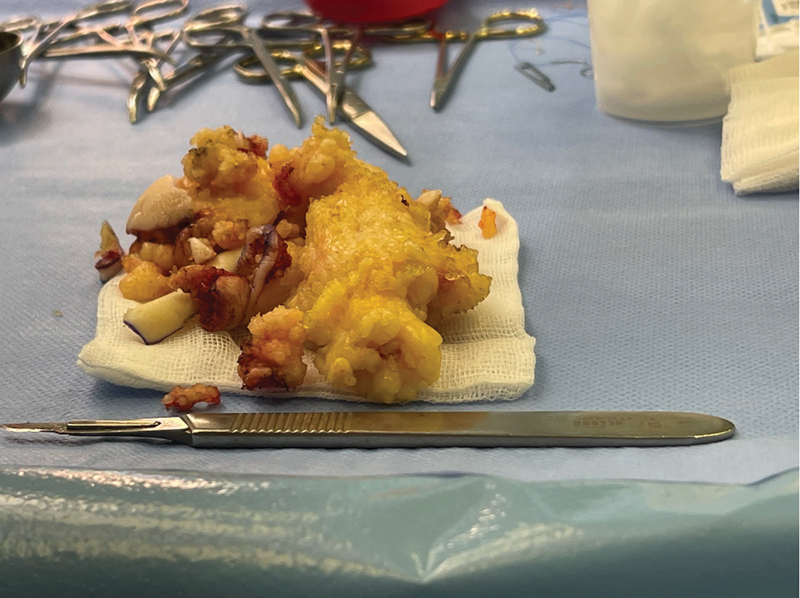
A photo showing the amount of tissue removed during foot surgery.

**Fig. 4:**
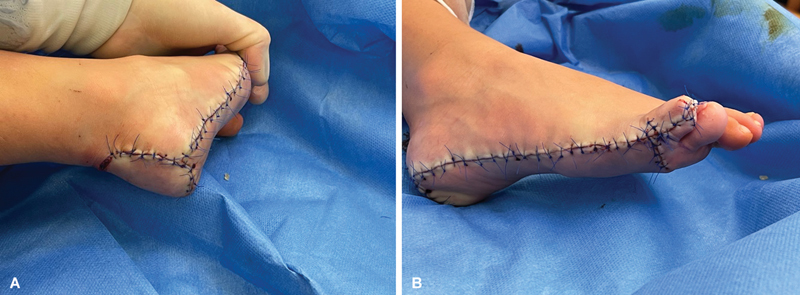
Photos ( **A, B** ) showing the result of the excess foot tissue removal surgery.

The patient underwent their initial surgery at our plastic surgery clinic at the age of 5 to address soft tissue hypertrophy in the foot ( Fig. 2 ). The procedure, performed under general anesthesia, was notable for its careful approach. Tissue dissection was performed without ischemia, despite the placement of a tourniquet on the thigh. A skin marker was used to mark the extent of skin excision on the lateral surface of the foot, including the fifth toe and the heel. Once the skin was exposed, masses of adipose tissue located between the dermis and muscle tissue were revealed. Removal of the overgrown adipose tissue along with excess skin from the lateral surface of the heel, the lateral surface of the foot and the fifth toe of the foot was performed ( Fig. 3 ). After proper hemostasis of the wound bed was achieved, the tissues were sutured, and a layered dressing was applied ( Fig. 4 ).

**Fig. 5:**
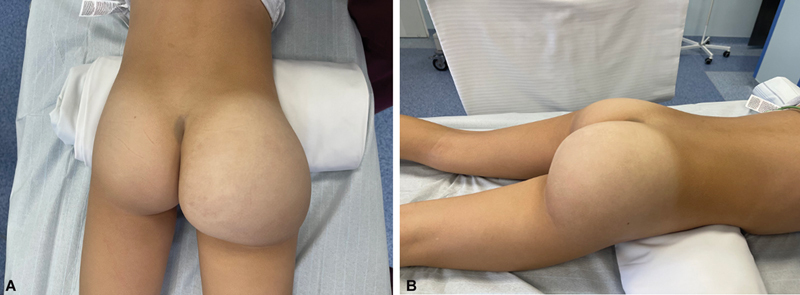
Photos ( **A, B** ) showing preoperative tissue overgrowth of the right buttock.

**Fig. 6:**
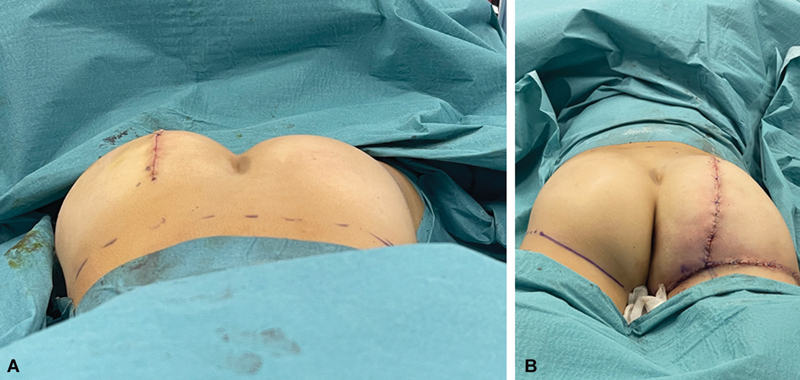
Photos ( **A, B** ) showing postoperative result of tissue reduction.

**Fig. 7:**
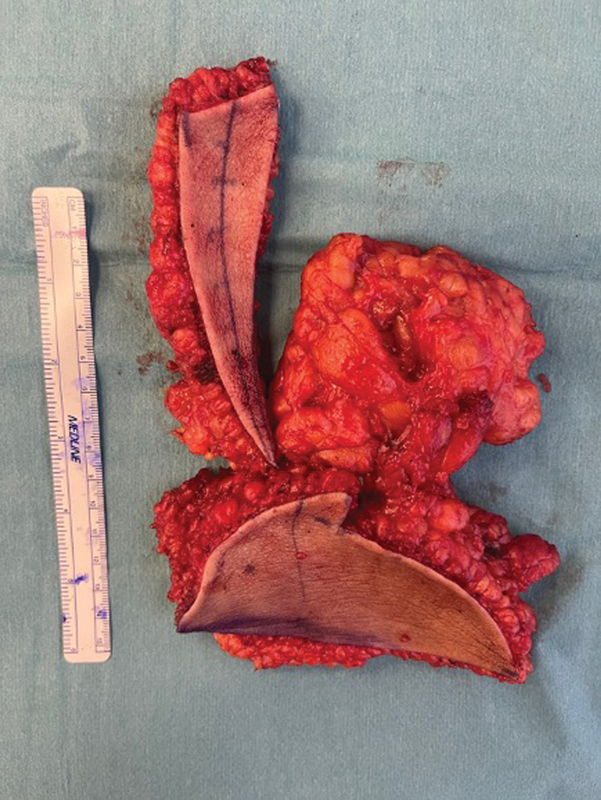
Photo showing the amount of tissue removed from the buttock during surgery.

A year after the reduction of the soft tissues of the foot, the child was qualified for the second procedure, the reduction of hypertrophy of the right buttock ( Fig. 5 ). Prior to the surgery, the child was prepared with oral laxatives to ensure the best comfort in the postoperative period. The operation was performed under general anesthesia with endotracheal intubation in the prone position. After the preparation of the surgical field, a longitudinal skin incision was planned in the medial quarter of the buttock and a transverse incision around the gluteal fold. After cutting the skin, masses of overgrown adipose tissue were exposed, which penetrated through the fascial septa toward the gluteal fissure. Reduction of the soft tissue hyperplasia consisted of an excision of a designated dermal fat tissue and removal of an overgrown adipose tissue from between the septum connecting the skin and the muscle fascia ( Fig. 6 ). The amount of removed soft tissues and the postoperative effect are shown in the pictures ( Fig. 7 ). No drain was left after the procedure. Good hemostasis was achieved during the operation. The wound was sutured in layers and a stabilizing dressing was applied. The child remained in the clinic for 2 days and was discharged in a good general and local condition after the dressing control.

**Fig. 8:**
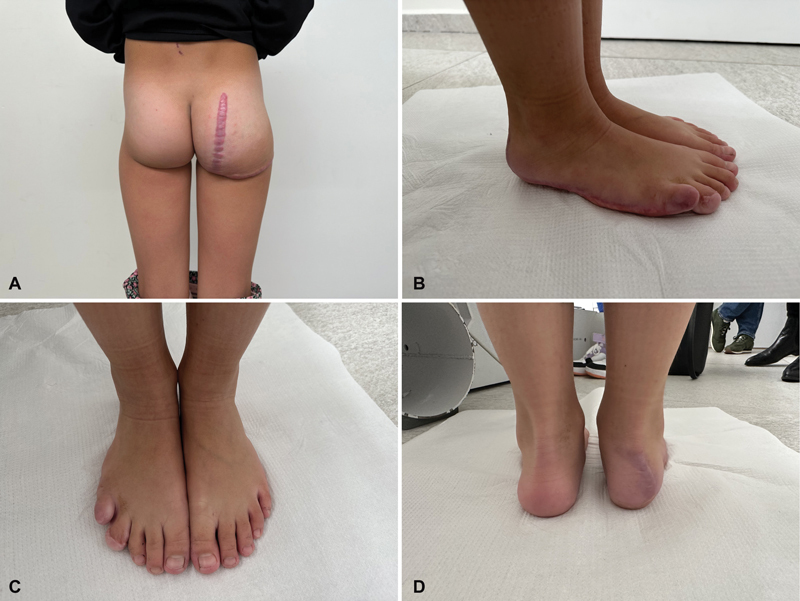
Follow-up photos taken 2 years after the foot surgery and 1 year after the buttock surgery. Photo ( **A** ) A photo showing the result of the buttock reduction surgery 1 year after procedure. No significant overgrowth has taken place during this 1-year period. The hypertrophic scar on the buttock is visible. Photos ( **B–D** ) showing the state of the patient's feet 2 years after the reduction surgery. No sign of overgrowth has been observed during the follow-up period.

Following both surgeries, the patient received acetaminophen (orally, four times a day, 15 mg/kg). The healing period was uneventful, devoid of complications, and the sutures were removed on the 14th day after the surgery. A significant hypertrophic tendency of scars was observed during the control visits, despite the use of compression therapy ( Fig. 8 ).

There have been no indications of overgrowth recurrence observed in the 2-year-period following the foot reduction surgery. The foot that underwent surgery continue to exhibit consistent proportions with the healthy one, considering the child's growth, without experiencing any unhealthy overgrowth of tissues since the operation ( Fig. 8 ).

The second buttock surgery, initially established in the treatment plan, is delayed by 2 years as the current size satisfies both parent and child. In the light of absence of relapse in foot overgrowth, its treatment was limited to observation only.

The overall quality of life, functional and aesthetic outcomes were evaluated 2 years after the foot surgery, using the Pediatric Quality of Life Inventory™ parent and patient report for young children aged 5 to 7 years ( Table 2 ). Total score of 97.5 for the parent report and 95 for the child report is very satisfactory and means a high health-related quality of life.

**Table 2:** Results of the Pediatric Quality of Life Inventory ^™^ Generic Core for young children aged 5 to 7 questionnaire

Dimension	Number of items	Score for parents report	Score for child report
Physical functioning	8	100	100
Emotional functioning	5	90	80
Social functioning	5	100	100
School functioning	5	100	100
Total score	23	97.5	95

Questionnaire evaluated four dimensions of patient's life in the past month. Scores are presented on the scale from 0 to 100. The higher the score, the better health-related quality of life.

## Discussion

PS is a rare asymmetrical and progressive hamartomatous syndrome that may affect many tissues and is barely noticeable at birth. In most cases, this condition initially manifests between 6 and 18 months of age in an irregular, progressively worsening manner, as exemplified by the patient in this report, who exhibited an increasing discrepancy between their body parts as the months passed. 10 This genetic disorder has highly variable clinical features, due to mosaic lesion distribution and sporadic occurrence, which can lead to misdiagnosis and confusion with other overgrowth syndromes. 1

We conducted a review of literature on this topic ( Table 3 ). 11 12 13 14 15 16 17 18 19 PS original studies and case reports were selected using PubMed, Embase, and Web of Science, these databases were searched for English language publications published from January 2014 to December 2023. The following search terms were used: (proteus [Title]) AND (syndrome [Title]). The inclusion criteria aimed to select PS studies that described skeletal and soft tissue malformations, limb deformities in cases with confirmed AKT1 mutation, that were treated (surgically and/or pharmacologically) and included a follow-up after treatment. Out of the 115 records initially identified, 80 full-text articles underwent eligibility and quality assessment after exclusion during the abstract review. Nine studies that met the inclusion criteria were included in the analysis. Some of the recent studies 11 12 13 14 17 show results of experimental gene targeting pharmaceutical therapy with sirolimus, ARQ 092, or miransertib-AKT1 inhibitors, originally designed to treat cancer. While these papers consistently highlight a reduction in area of cerebriform connective tissue nevus (CCTN), the improvement in the size of limb deformities has not been entirely satisfactory and patients might still require surgery to restore regular function.

**Table 3:** Literature review

Author, year	Study design	*N*	Sex	Age at the time of the treatment (years)	Age at the time of diagnosis (years)	Progression over time	Clinical features treated	Treatment	Follow-up
** Weibel et al, 2019 11**	Case report	1	F	9	2	Present	● Multiple lipomas on trunk ● Hemihypertrophy of right leg ● Plantar cerebriform connective tissue nevus (CCTN) ● Soft tissue abdominal swelling	Sirolimus	6 years follow-up: ● Reduction of 19,1% of plantar CCTN area ● Reduction in size of abdominal lipomas ● Skeletal deformations remained stable
** Ours et al, 2021 12**	Case report	1	M	18	6	Present	● Leg length discrepancy (LLD) ● Bilateral plantar CCTN ● Musculoskeletal pain of the lower back and lower limbs	Miransertib	48 weeks follow-up: ● Slowing of CCTN growth ● Less pain declared by the patient ● No improvement in leg length discrepancy
** Leoni et al, 2019 13**	Case report	1	F	17	1	Present	● Ipsilateral hand overgrowth ● Hyperostotic fusion of all cervical vertebrae, causing rotoscoliosis ● Plantar CCTN	Mirasertib	2 years follow-up: ● Reduction in CCTN size ● Increase in range of motion in forelimbs and joins (hand, spine, knees) ● Small reduction in size of the lesions on the hand and feet
** Lindhurst et al, 2015 14**	Case report	1	N/A	N/A	N/A	N/A	● Overgrowth of toes	ARQ 092	Phosphorylation of AKT was reduced in measured skin biopsies from overgrown toes.
** Crenshaw et al, 2018 15**	Original study	8	4 M, 4 F	9,4	N/A	Present	● LLD	Surgery	4.6 years follow-up: ● Improvement in LLD in all patients
** Modlin et al, 2022 16**	Case report	1	F	59	19	Present	● CCTN ● Papule on the left great toe	Surgery	5 years follow-up: ● Regrowth of CCTN, second surgery was needed ● Regrowth of left toe papule
** Keppler-Noreuil et al, 2022 17**	Original study	6	4 M 2 F	26,8	N/A	Present	● CCTN ● Bony overgrowth	Miransertib	12 months follow-up: ● Decrease in size of CCTN, softer and pliable ● No change in bony overgrowth
** Popescu et al, 2014 18**	Case report	1	M	5	1	Present	● Overgrowth of soft tissues of lower limb ● LLD	Surgery	Complete recurrence of LLD
** He and Zhao, 2020 19**	Case report	1	F	35	35	Present	● Overgrowth of the left foot	Surgery	Recurrence

Abbreviation: N/A, not available, not applicable.

There were several studies published concerning diagnostic criteria for PS. In 1999, Biesecker et al 20 developed phenotype-based diagnostic standards, which they subsequently updated and refined in 2006. 21 In 2011, Lindhurst et al 2 identified somatic activating mutation in AKT1, as a cause of PS, supporting the hypothesis of somatic mosaicism and implicating the activation of the phosphoinositide-3-kinase/protein kinse B (PI3K/AKT) pathway in the distinctive clinical manifestations of overgrowth and susceptibility to tumors in this condition. This finding discredited theory proposed earlier in the 21st century that the Phosphatase and TENsin (PTEN) hamartoma syndrome (also known as Cowden syndrome or Bannayan–Riley–Ruvalcaba syndrome) and PS had the same cause. 22 23 Current understanding reveals that loss-of-function mutations in PTEN (located on 10q23.3) activate AKT1, leading to certain shared features with PS. However, this activation results in a separate clinical phenotype. 2

In 2019, Sapp et al 24 introduced a novel diagnostic framework for identifying PS based on cases of 75 individuals. This system utilized a weighted, point-based approach to assess phenotypic attributes and subsequently incorporates potential molecular test results, classifying cases into one of the two designations: AKT1-related PS or AKT1-related overgrowth spectrum. We consider this system to be an effective diagnostic tool for PS. It accounts for a wide array of PS manifestations, recognizing the mosaic pleiotropic nature of the disorder, while also considering the presence of pathogenic *AKT1* gene variants. 24

In the case reported herein, the mutation of *ATK1* gene was confirmed by genetic testing. Patient was presented with asymmetric, disproportionate overgrowth of a lower limb, of a fast progressive fashion, as they required two reduction surgeries within a short time interval. There were manifestations of skeletal involvement in the overgrown feet. Dysregulated adipose tissue was found in the affected buttock, as well as vascular malformations. This exact clinical presentation has not been described yet in the literature, 1 2 3 4 5 6 7 8 9 10 20 21 22 23 24 25 26 which implies how this disease can manifest in many ways.

All pleiotropic disorders have phenotypic overlap, and it can be challenging to distinguish them. 24 PS must be differentiated from other overgrowth syndromes such as PTEN hamartoma tumor syndrome, Klippel–Trenaunay syndrome, and congenital lipomatous overgrowth, vascular malformations, epidermal nevis, spinal/skeletal anomalies/scoliosis (CLOVES) syndrome, 1 to avoid unnecessary testing and procedures. Table 4 summarizes the characteristics and distinguishing features of mentioned disorders. In 2007, Sapp et al 26 initially identified CLOVES syndrome in seven individuals previously diagnosed with PS. Distorted skeletal structures in CLOVES patients were primarily associated with major surgical interventions, contrasting with unoperated areas that remained unaffected. Hence surgeons should differentiate these two syndromes, as surgical procedures on the hands or feet in patients with CLOVES syndrome may induce skeletal overgrowth resembling those seen in PS. 1 26

**Table 4:** Characteristics and distinguishing features of overgrowth syndromes with overlapping phenotypes

Syndrome	OMIM	Mutation	Onset	Clinical characteristics
** CLOVES syndrome 25,26**	612918	PIK3CA	Prenatal	● Asymmetric body overgrowth with skeletal, vascular, visceral, and neural abnormalities ● Linear epidermal nevus along Blaschko's lines, vascular or neural structures with a hyperkeratotic and papillomatous surface ● Thoracic lipomatous hyperplasia ● Soft overgrowth of hands and feet that tends to form wrinkles ● No connective tissue nevus ● Mainly spinal, high flow arteriovenous malformations
** Klippel–Trenaunay syndrome 25,28**	149000	PIK3CA	Prenatal or postnatal	● Asymmetric limb hypertrophy and elongation (usually single lower extremity) ● Slow-flow vascular malformations involving lower and upper limbs and/or trunk often with persistent lateral embryologic veins ● Laterally located cutaneous hemangiomas (port-wine stains) ● Persistent embryonic lateral marginal vein of Servelle ● More often males than females
** PTEN hamartoma tumor syndrome 22,25**	601728	PTEN	Postnatal	● Asymmetric overgrowth of adipose tissue (lipomas) ● Development of noncancerous growths (hamartomas) in different areas of the body ● Term used to describe any patient with germline PTEN mutation (subtypes: Cowden Syndrome, Bannyan–Riley–Ruvalcaba syndrome and Proteus-like syndrome) ● Inherited in autosomal manner ● Increased risk of thyroid, skin, and colon cancer ● Intellectual disability, autism spectrum disorder, delay in motor development, macrocephaly ● Multiple mucocutaneous lesions

Abbreviation: PTEN, phosphatase and TENsin.

Early surgical procedures of distorted tissues are expected to not only improve aesthetic outcomes but also preserve proper psychological functioning. It is worth noting that there is a lack of evidence on this subject in the literature concerning PS. A study focused on the quality of life of children aged 2 to 12 years with capillary malformations of the lower limbs, particularly those affected by Klippel–Trenaunay syndrome, suggested that venous and orthopaedic complications in children were significantly associated with a decrease in their quality of life. 27 This observation remains significant as Klippel–Trenaunay syndrome may exhibit overlapping features with PS, including tissue overgrowth, which demands careful differentiation.

Another study, which enrolled adult patients, presented similar findings concerning overgrowth syndromes. Within this cohort, 95 patients were diagnosed with psychiatric conditions, 23.2% of the total, with depression (15.1%) and anxiety (5.1%) being the most identified conditions. 28

In our study, the strength of our foot reduction surgery lies in the application of a highly radical resection technique, potentially contributing to a lasting outcome. The buttock surgery involved tumor excision in three planes, reducing height, width, and projection. Notably, the procedure extended to subcutaneous removal of extensive adipose tissue clusters, compartmentalized by numerous fibrous septa, reaching toward the gluteal cleft. It enhanced aesthetic results and hygiene maintenance. Limitation is the challenging scarring process. The mid-foot and heel scars improved only after a year, while gluteal scars exhibited excessive growth despite compression therapy, remaining firm and broad. We expect a future relapse of the overgrowth in various body areas, due to progressive nature of the disorder, which is why our patient stays under observation.

### Conclusion

Currently there is no effective treatment for this syndrome, however given its monogenic nature, it is an excellent candidate for targeted therapy. We note that the extreme rarity of this disorder limits the researchers' opportunities for finding a favorable pharmacological cure. Considering our experience, we can presume that highly radical resection of pathological tissues may contribute to a longer lasting outcome. This is the first case report of this disease, explaining in such details the surgical approach, followed by a successful long-lasting result in this area of the body. We conclude that early diagnosis of patients with PS and subsequent surgical treatment may improve their quality of life and avoid social stigma.
